# Hygroscopic dilators vs balloon catheter ripening of the cervix for induction of labor in nulliparous women at term: Retrospective study

**DOI:** 10.1371/journal.pone.0189665

**Published:** 2017-12-22

**Authors:** Ryosuke Shindo, Shigeru Aoki, Naohiro Yonemoto, Yuriko Yamamoto, Junko Kasai, Michi Kasai, Etsuko Miyagi

**Affiliations:** 1 Perinatal Center for Maternity and Neonate, Yokohama City University Medical Center, Yokohama, Japan; 2 Department of Biostatistics and Epidemiology, Yokohama City University Graduate School of Medicine and University Medical Center, Yokohama, Japan; 3 Department of Obstetrics and Gynecology, Yokohama City University School of Medicine, Yokohama, Japan; Indiana University School of Medicine, UNITED STATES

## Abstract

**Objective:**

To compare the efficacy and safety of hygroscopic dilators and balloon catheters for ripening of the cervix in induction of labor.

**Study design:**

This retrospective, observational study used data from the Successive Pregnancy Birth Registry System of the Japan Society of Obstetrics and Gynecology from 2012 to 2014. Nulliparous women in whom labor was induced by mechanical methods of cervical ripening at term were enrolled. The eligible women were divided into dilator, balloon <40 mL, balloon ≧40 mL, and overlapping groups.

**Results:**

The groups included 4645, 4100, 6615, and 1992 women, respectively. In the overlapping group, which included the women in whom delivery was most difficult, the vaginal delivery rate was lower and the intrauterine infection and neonatal mortality rates were higher than those in the dilator group. No difference in the vaginal delivery rate was observed among the dilator, balloon <40 mL, and balloon ≧40 mL groups (74.6%, 72.3%, and 73.8%, respectively; p>0.05). The vaginal instrumental delivery rate was higher in the two-balloon groups than in the dilator group. The volume of intrapartum hemorrhage was lowest in the dilator group. No significant difference in the frequencies of uterine rupture and intrauterine infection were observed among the dilator and two-balloon groups. With regard to neonatal outcomes, the frequency of a low Apgar score was statistically significantly lower in the dilator group than in the two-balloon groups. Moreover, the frequency of neonatal death tended to be lower in the dilator group than in the two-balloon groups.

**Conclusion:**

With regard to cervical ripening for labor induction in nulliparous women at term, the vaginal delivery rate on using a dilator and on using a balloon seems to be equivalent. Concerning maternal complications and neonatal outcomes, cervical ripening with hygroscopic dilators in labor induction might be safer.

## Introduction

Two major iatrogenic methods for cervical ripening in labor induction are (1) mechanical, using a hygroscopic dilator or balloon catheter and (2) pharmacologic, such as transvaginal administration of prostaglandins. Many trials comparing balloon catheters and prostaglandins have shown that the use of balloon catheters is as effective as administration of prostaglandins [1–3]. A 2012 Cochrane Review [2] reported that compared to pharmacological methods, these mechanical methods are associated with a similar rate of cesarean section and a lower risk of uterine hyperstimulation. The usefulness and safety of the mechanical methods have been demonstrated.

Mechanical methods include insertion of a balloon catheter or placement of a hygroscopic dilator, of which the former is more commonly applied. In fact, balloon catheters were used in the majority of previous trials comparing mechanical and pharmacologic methods [1–8]. While single and double balloon catheters are used, trials comparing these types have shown no substantial difference in clinical outcomes [3, 9]. The effects of different balloon sizes have also been studied [10, 11]. A trial comparing 30 mL and 60 mL balloons showed no differences in maternal and neonatal outcomes [10].

Hygroscopic dilators have been reported to be safe and effective in trials comparing them to pharmacologic methods [12, 13]. In fact, dilators are more commonly used for pregnancy termination at early stages than for labor induction at term. We have not found any large-scale trials comparing the use of a hygroscopic dilator and other modalities for labor induction.

In Japan, because the National Health Insurance System does not approve transvaginal administration of prostaglandin E2 and other drugs for labor induction, mechanical methods are applied to induce cervical ripening in all cases. In addition to balloon catheters, hygroscopic dilators are also widely used. This study aimed to compare hygroscopic dilators and balloon catheters to evaluate their efficacy and safety for labor induction.

## Materials and methods

This study, which is a retrospective observational study using data from the Successive Pregnancy Birth Registry System of the Japan Society of Obstetrics and Gynecology (JSOG), was conducted after receiving approval from the ethics committee of the Yokohama City University Medical Center. Approximately 280 secondary and tertiary hospitals participate in the JSOG registry system, which contains information on successive deliveries occurring at 22 weeks of gestation or later. Each year, approximately 150,000 cases are entered into the registry system, accounting for approximately 15% of all deliveries in Japan. The data have been anonymized to prevent identification of individuals. Data collected between 2012 and 2014 were used in this study.

The inclusion criteria for participants were nulliparous women who underwent labor induction by mechanical methods of cervical ripening and delivered on or after gestational week 37. The exclusion criteria were non-cephalic presentation of a fetus, multiple pregnancies, and missing data.

Eligible women selected according to the above criteria were divided into 4 groups according to the mechanical method used for cervical ripening: 1) the dilator group, in which only a hygroscopic dilator was used; 2) the balloon < 40 mL group, in which only a < 40 mL balloon catheter was used; 3) the balloon ≥ 40 mL group, in which only a ≥ 40 mL balloon catheter was used; and 4) the overlapping group, in which multiple methods were performed in combination. Regardless of the combination or order of mechanical methods, the overlapping group included women who had undergone labor induction with 2 or all of the following 3 devices: a hygroscopic dilator, a < 40 mL balloon catheter, and a ≥ 40 mL balloon catheter.

The 4 groups were compared by characteristics including use of uterine stimulants, maternal age at delivery, maternal height at delivery (cm), maternal weight at delivery (kg), body mass index (BMI) at delivery, gestational age (week), baby’s weight (g), and baby’s sex. The primary outcome was the vaginal delivery rate. Secondary outcomes were vaginal instrumental delivery rate, intrapartum hemorrhage, postpartum hemorrhage (PPH), uterine rupture, intrauterine infection (clinical chorioamnionitis), maternal death, low umbilical artery pH (UApH) of < 7.1, low Apgar score (APS) of < 7 at 5 minutes, umbilical cord prolapse, and neonatal death. Intrapartum hemorrhage was defined as blood loss in the 2 hours after delivery, with PPH as blood loss of ≥ 500 mL in women undergoing vaginal delivery and ≥ 1000 mL in those undergoing cesarean section.

SPSS Statistics software version 23 (IBM Corp., Armonk, NY, USA) was used for statistical analyses. The characteristics of each group were expressed as mean (±standard deviation [SD]) or frequency (%) and analyzed using analysis of variance and the Tukey or chi-square test.

The primary and secondary outcomes were analyzed using multivariate logistic regression to calculate adjusted OR (AOR), and 95% confidence interval (CI) of the dilator group compared to the other 3 groups. Intrapartum hemorrhage was analyzed using multiple regression analysis to calculate adjusted regression coefficient (β) of the dilator group compared to the other 3 groups. Multivariate analyses were adjusted for maternal height at delivery, maternal weight at delivery, maternal age at delivery, baby’s weight, and baby’s sex. A significance level (*p*) of < 0.05 was considered to indicate a significant difference.

## Results

**Fig 1:**
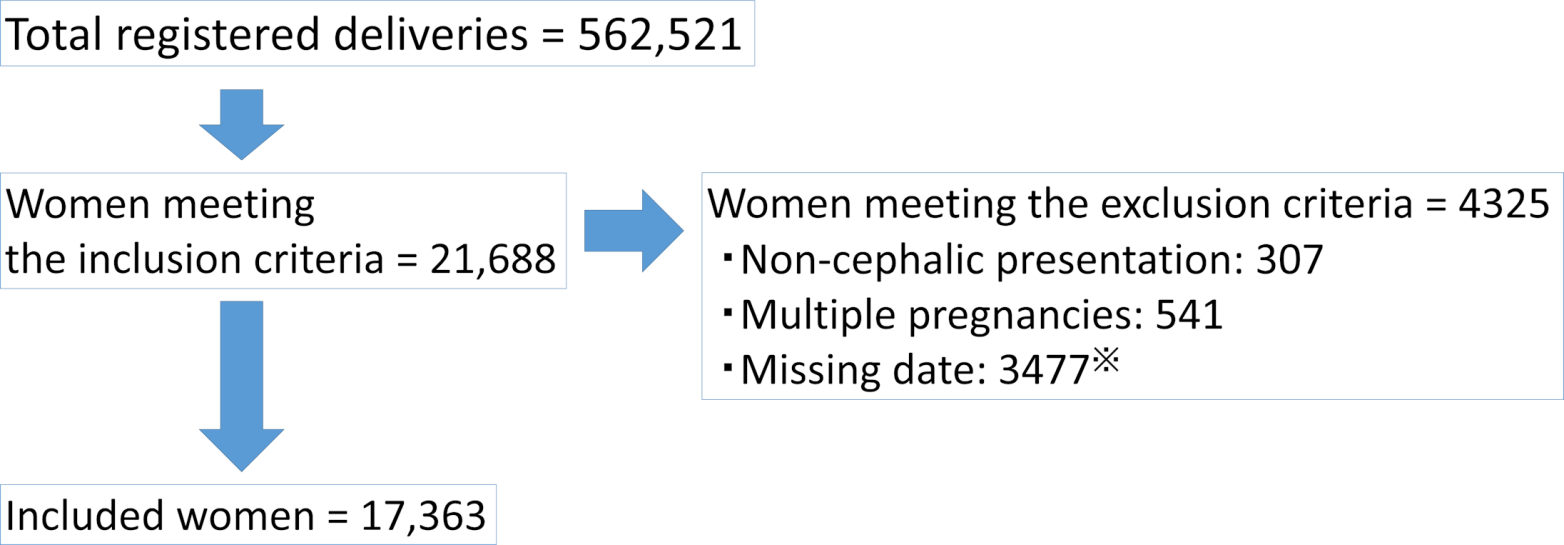
Flow chart for selection of eligible subjects. ※ Missing data: Women with missing or apparently incorrect data were excluded. The breakdown of excluded women is summarized as follows (including duplicates):
No data on the use of mechanical methods of cervical ripening: 379Maternal height: (no data and outside of a range of 120–200 cm) total 1301Maternal weight at delivery: (no data and outside of a range of 25–300 kg) total 2469Maternal age at delivery: (no data and outside of a range of 10–60 years: 0) total 25Neonatal weight at birth: (no data and outside of a range of 1000–6000 g) total 42Volume of intrapartum hemorrhage: (no data and <10 g: 15) total 115Apgar score at 5 minutes: no data: 37Umbilical artery pH: outside of a range of 6.0–8.0: 214 (Because umbilical artery pH is difficult to measure in some conditions, women without this value were included).

The total number of deliveries registered was 562,521. The inclusion criteria were met by *21*,*688* women; *4325* were excluded, for a final total of *17*,*363* women (Fig 1). There were *4650 (26*.*8%*) women in the dilator group, *4103* (*23*.*6%*) in the balloon < 40 mL group, *6616* (*38*.*1%*) in the balloon ≥ 40 mL group, and *1994* (*11*.*5%*) in the overlapping group. Labor induction was most frequently performed with a ≥ 40 mL balloon catheter alone.

Table 1 shows the maternal, pregnancy, and neonatal characteristics of each group. The rates of concomitant uterine stimulant use were 88.7%, 84.0%, 90.2%, and 92.6%, respectively, exceeding 80% in all groups because the use of hygroscopic dilators or balloon catheters is primarily for cervical ripening and to achieve a favorable Bishop score for successful labor induction.

**Table 1:** Maternal, pregnancy, and neonatal characteristics of each group.

		1) dilator	2) balloon <40	3) balloon ≧40	4) overlapping	
		N = 4650	N = 4103	N = 6616	N = 1994	p
use of uterine stimulants	n (%)	4126 (88.7%)	3445 (84.0%)	5968 (90.2%)	1846 (92.6%)	<0.01
maternal height (cm)	mean±SD	158.7±5.6	158.5±5.6	158.3±5.6	158.4±5.6	0.01
maternal weight (kg)	mean±SD	64.0±10.2	64.9±11.2	66.0±11.3	66.2±11.3	<0.01
maternal BMI	mean±SD	25.4±3.8	25.9±4.2	26.3±4.2	26.4±4.1	<0.01
maternal age	mean±SD	32.7±5.4	32.0±5.8	31.6±5.8	32.4±6.0	0.02
gestational age (week)	mean±SD	39.7±1.4	40.0±1.3	40.0±1.3	40.0±1.4	0.03
baby's weight	mean±SD	2998±431	3053±452	3055±454	3038±477	<0.01
baby's sex (boy)	n (%)	2311 (49.8%)	1977 (48.2%)	3250 (49.1%)	945 (47.4%)	0.27

Table 2 shows the delivery outcomes. No difference in the vaginal delivery rate was observed among the dilator, the balloon < 40ml and the balloon ≥ 40 mL groups (*balloon < 40ml group*: *74*.*6% vs 72*.*3%*, *AOR 0*.*91*, *95% CI 0*.*83–1*.*01*, *balloon* ≧40ml group: vs 72.8%, AOR 1.01, 95% CI 0.93–1.11). The rate in the overlapping groups was statistically significantly lower than the rate in the dilator group (*74*.*6% vs 63*.*6%*, *AOR 0*.*63*, *95% CI 0*.*56–0*.*71*).

**Table 2:** Delivery outcome.

		Dilator n = 4650	balloon<40 n = 4103	p	balloon≧40 n = 6616	p	overlapping n = 1994	p
vaginal delivery	Frequency	3467 (74.6%)	2967 (72.3%)	-	4883 (73.8%)	-	1269 (63.6%)	-
	AOR^*1^ *(95% C*.*I)*	1	*0*.*91 (0*.*83–1*.*01)*	*0*.*07*	*1*.*01(0*.*93–1*.*11)*	*0*.*41*	*0*.*63 (0*.*56–0*.*71)*	*<0*.*01*
vaginal instrumental delivery	Frequency(%)	725 (15.4%)	723 (17.4%)	-	1164 (17.6%)	-	339 (17.0%)	-
	AOR^*1^ *(95% C*.*I)*	*1*	*1*.*20 (1*.*07–1*.*34)*	*<0*.*01*	*1*.*24 (1*.*12–1*.*38)*	*<0*.*01*	*1*.*17 (1*.*02–1*.*35)*	*0*.*03*
intrapartum hemorrhage	mean±SD (g)	573±469	628±454	-	*604±441*	*-*	678±630	-
	β^*2^ *(95% C*.*I)*	1	46 (27–66)	*<0*.*01*	*22 (5–40)*	*0*.*01*	91 (67–115)	<0.01
Postpartum hemorrhage	frequency(%)	1527 (32.8%)	1591 (38.8%)	-	2446 (37.0%)	-	766 (38.4%)	-
	AOR^*1^ *(95% C*.*I)*	*1*	*1*.*25 (1*.*14–1*.*37)*	*<0*.*01*	*1*.*16 (1*.*01–1*.*26)*	*<0*.*01*	*1*.*23 (1*.*10–1*.*37)*	*<0*.*01*
uterine rupture	frequency(%)	2 (<0.1%)	1 (<0.1%)	-	1 (<0.1%)	-	1 (<0.1%)	-
	AOR^*1^ *(95% C*.*I)*	*1*	*0*.*56 (0*.*05–6*.*25)*	*0*.*64*	*0*.*31 (0*.*03–3*.*57)*	*0*.*35*	*1*.*08 (0*.*10–11*.*94)*	*0*.*95*
intrauterine infection	frequency(%)	61 (1.3%)	48 (1.2%)	-	104 (1.6%)	-	55 (2.8%)	-
	AOR^*1^ *(95% C*.*I)*	*1*	*0*.*83 (0*.*56–1*.*21)*	*0*.*32*	*1*.*08 (0*.*79–1*.*49)*	*0*.*63*	*1*.*93 (1*.*33–2*.*79)*	*<0*.*01*
Umbilical artery pH <7.1	frequency(%)	56 (1.3)	49 (1.3%)	-	116 (1.9%)	-	30 (1.6%)	-
	AOR^*1^ *(95% C*.*I)*	*1*	*0*.*99 (0*.*67–1*.*46)*	*0*.*92*	*1*.*46 (1*.*06–2*.*02)*	*0*.*02*	*1*.*21 (0*.*78–1*.*90)*	*0*.*40*
Apgar score at 5min < 7	frequency(%)	45 (1.0%)	56 (1.4%)	-	92 (1.4%)	-	29 (1.5%)	-
	AOR^*1^ *(95% C*.*I)*	*1*	*1*.*49 (1*.*00–2*.*21)*	*<0*.*05*	*1*.*49 (1*.*04–2*.*15)*	*0*.*03*	*1*.*50 (0*.*94–2*.*40)*	*0*.*09*
neonatal death	frequency(%)	13 (0.3%)	21 (0.5%)		32 (0.5%)	-	14 (0.7%)	-
	AOR^*1^ *(95% C*.*I)*	*1*	*1*.*99 (0*.*99–3*.*99)*	*0*.*05*	*1*.*87 (0*.*97–3*.*58)*	*0*.*06*	*2*.*42 (1*.*13–5*.*20)*	*0*.*02*
umbilical cord prolapse	frequency(%)	0	2 (<0.1%)	-	3 (0.1%)	-	1 (0.1%)	-

*1 Adjusted odds ratio adjusted for maternal age at delivery, maternal height at delivery, maternal weight at delivery, baby’s weight, and baby’s sex. Values in parentheses are 95% confidence interval.

*2 β = regression coefficient adjusted for maternal age at delivery, maternal height at delivery, maternal weight at delivery, baby’s weight, and baby’s sex. Values in parentheses are 95% confidence interval.

The vaginal instrumental delivery rate was statistically significantly lower in the dilator group than in the other 3 groups. (15.4% vs 17.4%, 17.6%, 17.0%. AOR (95% CI) 1.20 (1.07–1.34), 1.24(1.12–1.38), 1.17(1.02–1.35))

The volume of intrapartum hemorrhage (mean ± SD) was *573 g* (± *469 g*) in the dilator group, *628 g* (± *454 g*) in the balloon < 40 mL group, *604 g* (± *441 g*) in the balloon ≥ 40 mL group, and *678 g* (*± 630 g*) in the overlapping group; it was lowest in the dilator group. The frequency of PPH was lowest in the dilator group (*32*.*8% vs 38*.*8% vs 37*.*0% vs 38*.*4%*). The frequency of intrauterine infection was not statistically significant different between the dilator group and the balloon < 40 mL or balloon ≥ 40 mL group, but was statistically significantly higher in the overlapping group. No statistically significant difference was observed in the frequency of uterine rupture. No maternal death occurred in any group.

As for neonatal outcomes, the frequency of low UApH was highest in the balloon ≥ 40 ml group. Low APS was statistically significantly lower in the dilator group than the balloon <40ml and the balloon ≧40 mL groups. The frequency of neonatal death was statistically significantly higher in the overlapping group than in the other groups. Moreover, despite the lack of a statistically significant difference, the frequency was lower in the dilator group than in the two balloon groups. The incidence of umbilical cord prolapse was low and observed in 6 women, including 2 in the balloon < 40 mL group, 3 in the balloon ≥ 40 mL group, and 1 in the overlapping group. This condition was observed only in women undergoing labor induction with balloon catheters, while it did not occur in any women in the dilator group.

## Discussion

No difference in the vaginal delivery was observed among the dilator, the balloon <40ml and the balloon ≥ 40 mL groups. Although no trials comparing hygroscopic dilators and balloon catheters directly have been reported, a randomized controlled trial conducted in 2011 comparing balloon catheters and prostaglandin E2 gel (PROBAAT trial) showed that vaginal delivery rates were 77% in women undergoing labor induction with a balloon catheter and 80% in those undergoing labor induction with prostaglandin E2 gel [1]. The rate of 74.6% obtained in the dilator group of our study. Since this study included only nulliparous women, the vaginal delivery rate was slightly lower than that in the aforementioned randomized, controlled trial of multiparous women. However, the rates in reports on labor induction by other mechanical methods ranged from 64.8% to 71% [14–16], and they were almost comparable to the rate in our study. The volume of intrapartum hemorrhage was smaller and the frequency of PPH was lower in the dilator group than in the other groups. Neonatal outcomes were favorable, and umbilical cord prolapse did not occur in this group.

The volume of intrapartum hemorrhage in the dilator group was significantly smaller and frequency of PPH was also lower than in the other 3 groups. It may be associated with the rate of vaginal instrumental delivery. Vaginal instrumental delivery increases the volume of intrapartum hemorrhage [17]. In this study, because the dilator group had the lowest vaginal instrumental delivery rate, the volume of intrapartum hemorrhage was small and the frequency of PPH was low.

As for neonatal outcomes, the frequency of neonatal death was statistically significantly higher in the overlapping group than in the other groups. Moreover, despite the lack of a statistically significant difference, the frequency was lower in the dilator group than in the two balloon groups. The frequencies of low APS were statistically significantly lower in the dilator group than in the balloon groups. The frequencies of low UApH was highest in the balloon ≧40ml group.

In the balloon <40 and ≧40 mL groups, the instrument delivery rate was higher than that in the dilator group, the amount of intrapartum hemorrhage was increased, and the neonatal prognosis was poor. We speculate that these were because of hyperstimulation caused by concurrent use of a balloon catheter and uterine stimulants [13]. Hyperstimulation might induce a non-reassuring fetal status (NRFS), which causes instrumental delivery. Increased instrumental delivery could increase maternal hemorrhage. This might be based on the guidelines in Japan regarding the concurrent use of cervical ripening agents and uterine stimulants. Concurrent use of a hygroscopic dilator and uterine stimulants is prohibited in Japan; when they are used in combination, the hygroscopic dilator must be removed before administration of uterine stimulants. Balloon catheters may, however, be used in combination with uterine stimulants after 1 hour of catheterization unless cervical ripening is extremely poor [13]. Because of such a rule, hyper-stimulation is likely to occur in using balloon catheters, which may affect the results of this research.

Although we were unable to perform statistical analysis of umbilical cord prolapse because of low frequency, it is noteworthy that this condition occurred only in women undergoing labor induction with balloon catheters. The use of balloon catheters has previously been reported to increase the risk of umbilical cord prolapse [18–21]. In labor induction with a balloon catheter, because a space is created between the uterine os and the fetal head, Umbilical cord prolapse may be caused without fore-lying and occult prolapse of umbilical cord at the time of insertion. Despite the low frequency, umbilical cord prolapse is a serious complication for neonates and requires attention. According to the results of this study, this complication did not occur in any women in the dilator group. This can be a great advantage for cervical ripening with a hygroscopic dilator.

No difference in the frequency of intrauterine infection was observed between the dilator group and the balloon < 40 mL or balloon ≥ 40 mL group. Intrauterine infection has been reported as a side effect of all mechanical methods [14, 22, 23].Use of balloon catheters has been associated with a higher risk of maternal and neonatal infection than administration of prostaglandins, whereas intrauterine infection has been reported to have no impact on maternal or neonatal outcomes [1, 11, 24, 25]. This study showed no difference in the frequency of intrauterine infection between the dilator group and the balloon < 40 mL or balloon ≥ 40 mL groups, confirming that the mechanical method using a hygroscopic dilator is no more likely to cause infection than that using balloon catheters.

This study has several limitations. First, because it was a retrospective, multicenter study, there is some concern that the induction methods would greatly differ between nulliparous and multiparous women, depending on hospital policies. Thus, since this study included only nulliparous women, no multiparous women were examined. Second, the JSOG registry system includes many pregnant women with missing data. Third, because this system does not provide data on the concurrent use of uterine stimulants in women undergoing labor induction with balloon catheters, the labor duration, and reason for forced delivery (instrumental vaginal delivery or cesarean section), we could not consider these factors. Fourth, because balloon catheters could not be inserted without the uterine os dilated to some extent, there might have been a bias towards the use of a hygroscopic dilator in women with poorer cervical ripening. The results of this observational study need to be validated in large-scale randomized controlled trials comparing hygroscopic dilators and balloon catheters.

## Conclusion

This study’s findings demonstrated that the vaginal delivery rate for cervical ripening with the hygroscopic dilator was comparable to that for cervical ripening with balloon catheters. Moreover, the number of women undergoing vaginal instrumental delivery was smaller in the dilator group than in the two balloon groups. Furthermore, the volume of intrapartum hemorrhage was smaller and the frequency of PPH was lower in the dilator group than in the other groups. Neonatal outcomes were favorable, and umbilical cord prolapse did not occur in this the dilator group. The mechanical methods of cervical ripening have recently been reevaluated in terms of safety and low cost, and balloon catheters are widely used. However, cervical ripening with a hygroscopic dilator appears to be a safer method.

## References

[1] Jozwiak M, Oude Rengerink K, Benthem M, van Beek E, Dijksterhuis MG, de Graaf IM, et al Foley catheter versus vaginal prostaglandin E2 gel for induction of labour at term (PROBAAT trial): an open-label, randomised controlled trial. *Lancet*. 2011; 378(9809): 2095–103. doi: 10.1016/S0140-6736(11)61484-0 22030144 10.1016/S0140-6736(11)61484-0

[2] Jozwiak M, Bloemenkamp KW, Kelly AJ, Mol BW, Irion O, Boulvain M. Mechanical methods for induction of labour. *Cochrane Database Syst Rev*. 2012;3: CD001233 10.1002/14651858.CD001233.pub2 22419277

[3] Chen W, Xue J, Peprah MK, Wen SW, Walker M, Gao Y, et al A systematic review and network meta-analysis comparing the use of Foley catheters, misoprostol, and dinoprostone for cervical ripening in the induction of labor. *BJOG*. 2016; 123(3): 346–54. doi: 10.1111/1471-0528.13456 26538408 10.1111/1471-0528.13456

[4] Kosinska-Kaczynska K, Ciechanowicz P, Saletra A, Szymusik I, Wielgos M. Two methods of cervix ripening: intracervical Foley catether and dinoprostone—which one is actually more efficient? *Neuro Endocrinol Lett*. 2015;36(3): 257–61. 26313393

[5] Rath W, Kehl S. The renaissance of transcervical balloon catheters for cervical ripening and labour induction. *Geburtshilfe Frauenheilkd*. 2015;75(11): 1130–9. doi: 10.1055/s-0035-1558094 26719596 10.1055/s-0035-1558094 PMC4678052

[6] Wang H, Hong S, Liu Y, Duan Y, Yin H. Controlled-release dinoprostone insert versus Foley catheter for labor induction: a meta-analysis. *J Matern Fetal Neonatal Med*. 2016;29(14): 2382–8. doi: 10.3109/14767058.2015.1086331 26427519 10.3109/14767058.2015.1086331

[7] Pennell CE, Henderson JJ, O’Neil MJ, McChlery S, Doherty DA, Dickinson JE. Induction of labor in nulliparous women with an unfavorable cervix: a randomised controlled trial comparing double and single balloon catheters and PGE2 gel. *BJOG*. 2009;116(11): 1443–52. doi: 10.1111/j.1471-0528.2009.02279.x 19656148 10.1111/j.1471-0528.2009.02279.x

[8] Salim R, Zafran N, Nachum Z, Garmi G, Kraiem N, Shalev E. Single-balloon compared with double-balloon catheters for induction of labor: a randomized controlled trial. *Obstet Gynecol*. 2011;118(1): 79–86. doi: 10.1097/AOG.0b013e318220e4b7 21691166 10.1097/AOG.0b013e318220e4b7

[9] Levy R, Kanengiser B, Furman B, Ben Arie A, Brown D, Hagay ZJ. A randomized trial comparing a 30-ml and an 80-ml Foley catheter balloon for preinduction cervical ripening. *Am J Obstet Gynecol*. 2004;191(5): 1632–6. doi: 10.1016/j.ajog.2004.03.033 15547534 10.1016/j.ajog.2004.03.033

[10] Delaney S, Shaffer BL, Cheng YW, Varqas J, Sparks TN, Paul K, et al Labor induction with a Foley balloon inflated to 30 mL compared with 60 mL; a randomized controlled trial. *Obstet Gynecol*. 2010;115(6): 1239–45. doi: 10.1097/AOG.0b013e3181dec6d0 20502296 10.1097/AOG.0b013e3181dec6d0

[11] Chua S, Arulkumaran S, Vanaja K, Ratnam SS. Preinduction cervical ripening: prostaglandin E2 gel vs hygroscopic mechanical dilator. *J Obstet Gynaecol Res*. 1997;23(2): 171–7. 9158305 10.1111/j.1447-0756.1997.tb00828.x

[12] Gilson GJ, Russell DJ, Izquierdo LA, Qualls CR, Curet LB. A prospective randomized evaluation of a hygroscopic cervical dilator, Dilapan, in the preinduction ripening of patients undergoing induction of labor. *Am J Obstet Gynecol*. 1996;175(1): 145–9. 8694040 10.1016/s0002-9378(96)70264-8

[13] Minakami H, Maeda T, Fujii T, Hamada H, Iitsuka Y, Itakura A, et al Guidelines for obstetrical practice in Japan: Japan Society of Obstetrics and Gynecology (JSOG) and Japan Association of Obstetricians and Gynecologists (JAOG) 2014 edition. *J Obstet Gynaecol Res*. 2014;40(6): 1469–99. doi: 10.1111/jog.12419 24888907 10.1111/jog.12419

[14] Krammer J, Williams MC, Sawai SK, O'Brien WF. Pre-induction cervical ripening: a randomized comparison of two methods. *Obstet Gynecol*. 1995;85(4): 614–8. doi: 10.1016/0029-7844(95)00013-H 7898843 10.1016/0029-7844(95)00013-H

[15] Pettker Christian M., Pocock Sean B., Dorothy P. Smok, Lee SM, Devine PC. Transcervical Foley Catheter with and without oxytocin for cervical ripening. Obstet Gynecol. 2008; 111 (6): 1320–6. doi: 10.1097/AOG.0b013e31817615a0 18515515 10.1097/AOG.0b013e31817615a0

[16] Mackeen A.Dhanya, Walker La Toya, Ruhstaller Kelley, Schuster M, Sciscione A. Foley Catheter vs Prostaglandin as Ripening Agent in Pregnant Women With Premature Rupture of Membranes. J Am Oateopath Assoc. 2014; 114 (9): 686–692. 10.7556/jaoa.2014.137 25170038

[17] Gardella Carolyn, Taylor Melanie, Benedetti Thomas, Hitti J, Critchlow C. The effect of sequential use of vacuum and forceps for assisted vaginal delivery on neonatal and maternal outcomes. Am J Obstet Gynecol. 2001; 185: 896–902. doi: 10.1067/mob.2001.117309 11641674 10.1067/mob.2001.117309

[18] Hasegawa J, Sekizawa A, Ikeda T, Koresawa M, Ishiwata Y, Kawabata M, et al; Japan Association of Obstetricians and Gynecologists. The use of balloons for uterine cervical ripening is associated with an increased risk of umbilical cord prolapse: population based questionnaire survey in Japan. *BMC Pregnancy Childbirth*. 2015;15: 4 doi: 10.1186/s12884-015-0432-4 25927949 10.1186/s12884-015-0432-4 PMC4310172

[19] Hasegawa J, Ikeda T, Sekizawa A, Ishiwata I, Kinoshita K; Japan Association of Obstetricians and Gynecologists. Obstetric risk factors for umbilical cord prolapse: a nationwide population-based study in Japan. *Arch Gynecol Obstet*. 2016;294(3): 467–72. doi: 10.1007/s00404-015-3996-3 26714678 10.1007/s00404-015-3996-3

[20] Yamada T, Cho K, Yamada T, Morikawa M, Minakami H. Labor induction by transcervical balloon catheter and cerebral palsy associated with umbilical cord prolapse. *J Obstet Gynaecol Res*. 2013;39(6): 1159–64. doi: 10.1111/jog.12036 23551955 10.1111/jog.12036

[21] Yamada T, Kataoka S, Takeda M, Kojima T, Yamada T, Morikawa M, et al Umbilical cord presentation after use of a trans-cervical balloon catheter. *J Obstet Gynaecol Res*. 2013;39(3): 658–62. doi: 10.1111/j.1447-0756.2012.02008.x 23003562 10.1111/j.1447-0756.2012.02008.x

[22] Heinemann J, Gillen G, Sanchez-Ramos L, Kaunitz AM. Do mechanical methods of cervical ripening increase infectious morbidity? A systematic review. *Am J Obstet Gynecol*. 2008;199(2): 177–87. doi: 10.1016/j.ajog.2008.05.005 18674661 10.1016/j.ajog.2008.05.005

[23] Jozwiak M, van de Lest HA, Burger NB, Dijksterhuis MG, De Leeuw JW. Cervical ripening with Foley catheter for induction of labor after cesarean section: a cohort study. *Acta Obstet Gynecol Scand*. 2014;93(3): 296–301. doi: 10.1111/aogs.12320 24354335 10.1111/aogs.12320

[24] McMaster K, Sanchez-Ramos L, Kaunitz AM. Evaluation of a transcervical Foley catheter as a source of infection: a systematic review and meta-analysis. *Obstet Gynecol*. 2015;126(3): 539–51. doi: 10.1097/AOG.0000000000001002 26244535 10.1097/AOG.0000000000001002

[25] Halem KV, Bakker JJ, Verhoeven CJ, Papatsonis DN, Oudqaarden ED, Janssen P, et al Does use of an intrauterine catheter during labor increase risk of infection? *J Matern Fetal Neonatal Med*. 2012;25(4): 415–8. doi: 10.3109/14767058.2011.582905 21649507 10.3109/14767058.2011.582905

